# Reliability and consistency of the Japanese version of the Primary Lateral Sclerosis Functional Rating Scale

**DOI:** 10.1186/s12883-024-03729-6

**Published:** 2024-08-13

**Authors:** Masaru Yanagihashi, Takehisa Hirayama, Mari Shibukawa, Junpei Nagasawa, Koji Fujita, Yuishin Izumi, Mitsuya Morita, Kota Bokuda, Kazushi Takahashi, Kazuaki Kanai, Naoki Atsuta, Yohei Iguchi, Masahisa Katsuno, Yoshitaka Murakami, Hiroshi Mitsumoto, Osamu Kano

**Affiliations:** 1 Department of Neurology, Toho University Faculty of Medicine, Tokyo, Japan; 2 Department of Neurology, Tokushima University Graduate School of Biomedical Sciences, Tokushima, Japan; 3 Rehabilitation Center, Jichi Medical University Hospital, Shimotsuke, Japan; 4 Department of Neurology, Tokyo Metropolitan Neurological Hospital, Tokyo, Japan; 5 Department of Neurology, Fukushima Medical University School of Medicine, Fukushima, Japan; 6 Department of Neurology, Aichi Medical University School of Medicine, Aichi, Japan; 7 Department of Neurology, Nagoya University Graduate School of Medicine, Nagoya, Japan; 8 Department of Neurology, Department of Clinical Research Education, Nagoya University Graduate School of Medicine, Nagoya, Japan; 9 Department of Medical Statistics, Toho University Faculty of Medicine, Tokyo, Japan; 10 Department of Neurology, Columbia University Irving Medical Center, New York, NY USA

**Keywords:** Primary lateral sclerosis, PLSFRS Japanese version, Face-to-face assessments

## Abstract

**Background:**

Primary lateral sclerosis (PLS) is an extremely rare condition; therefore, to date no clinical studies have been conducted. The Primary Lateral Sclerosis Functional Rating Scale (PLSFRS) was developed in the United States of America. The PLSFRS is a crucial assessment scale for international collaborative research and future clinical trials for PLS. It is useful for evaluating medical conditions through face-to-face assessments and telephone interviews such as when a face-to-face assessment is not possible due to disasters or the burden of hospital visits. This study assessed the reliability and consistency of in-person and telephone interviews using the Japanese version of the PLSFRS.

**Methods:**

We enrolled 19 Japanese patients who met the specific criteria for inclusion at the six collaborating institutions. The PLSFRS assessments were performed by two evaluators at defined time points and analyzed for intra-rater and inter-rater reliability and consistency between the in-person and telephone interviews.

**Results:**

The Japanese version of the PLSFRS was developed by a specialized company and translator, and modified to consider the Japanese lifestyle through a consensus among motor neuron specialists. The quadratic-weighted kappa coefficients for the intra-rater and the inter-rater agreement were substantial (intra-rater: 0.691-1.000, inter-rater: 0.634-1.000). Moreover, the intraclass correlation coefficient for the PLSFRS total score was 0.997 (95% confidence interval, 0.992–0.999).

**Conclusions:**

This study provides results regarding the Japanese version of the PLSFRS intra-rater and inter-rater reliability and consistency between in-person and telephone interviews.

**Supplementary Information:**

The online version contains supplementary material available at 10.1186/s12883-024-03729-6.

## Background

Primary lateral sclerosis (PLS) is a progressive neurodegenerative disease in which upper motor neurons are selectively damaged. PLS is rare, occurring in less than 5% of motor neuron diseases [1, 2]; therefore to date, no therapeutic agents or clinical trials have been evaluated. Since there have been no indicators to evaluate PLS, in 2020 a PLS Functional Rating Scale (PLSFRS) was developed in the United States of America, and the results were consistent with intra-rater, inter-rater, face-to-face visits, and telephone test-retest reliability during 1 year [3]. The PLSFRS is an essential tool for future international collaborative studies and clinical trials for PLS. Our study evaluated the reliability of the Japanese version of the PLSFRS and assessed the consistency between face-to-face evaluations and telephone interviews.

## Methods

### Institutional review board and volunteer participation

The study protocol was approved by the Ethics Committee of Toho University Faculty of Medicine, Tokyo, Japan (A21013, M20282), and other participating institutions (4058, rinfu 21 − 008, TS R03-017, ippan2021-318, 2021 − 0101). Written informed consent was obtained from all the patients. The PLSFRS was translated into Japanese by a specialized company and translator (2-14-10 Sotokanda Chiyoda-ku Tokyo 101 − 0021 Crimson Interactive Japan Co., ltd.), and it was further modified to account for the Japanese lifestyle based on consensus among the motor neuron specialists. Nineteen patients were enrolled in the study.

### Protocol and inclusion and exclusion criteria

The study and evaluation protocols are presented in Table 1. Face-to-face examinations and telephone interviews were conducted at intervals of at least 24 h but no more than 7 days. All evaluators for this study were board-certified neurologists and were trained appropriately before the evaluation. The inclusion and exclusion criteria are listed in Table 2. The patients were encouraged to state their condition, and the caregivers were advised not to make their interpretations. Participants were excluded if they withdraw consent, died, or were deemed inappropriate to continue in the study.

**Table 1:** Study protocol

Time	Baseline	4 weeks later		8 weeks later
Way to evaluate	Face-to-face	Face-to-face	Telephone	Face-to-face
Eligibility Assessment	P^†^			
Consent/Enrollment	P			
PLSFRS Evaluation	P	S^‡^	S	P

P^†^, primary evaluator; PLSFRS, Primary Lateral Sclerosis Functional Rating Scale; S^‡^, secondary evaluator

**Table 2:** Inclusion and exclusion criteria

Inclusion criteria	Exclusion criteria
• Japanese who are 20 years of age or older. • No family history or consanguinity. • No cognitive decline or hearing loss. • Unexplained upper motor neuron disorders^†^ in at least two of the three regions of the brain, upper limbs, and lower limbs regardless of the disease duration. • Normal needle electromyography^‡^, or no evidence of active lower motor neuron disorders, • Able to sign a consent form (if unable to sign due to muscle weakness in the upper extremities, or unable to respond due to dysarthria, writing on behalf of the patient was acceptable).	• Cognitive decline or hearing loss, including proxy. • If consent to this research was not obtained. • Other cases the researcher determined inappropriate, such as the patient being unable to express intentions accurately.

^†^Upper motor neuron dysfunction included spasticity and associated weakness, pathological hyperreflexia that is Hoffman’s’ sign and bilateral extensor toe responses, pseudobulbar affect

^‡^Normal needle electromyography or the absence of active lower motor neuron degeneration that minimally increased insertional activity and positive sharp waves or fibrillation potentials in extremity muscles

### Statistical analyses

We conducted statistical analyses of the PLSFRS to assess intra-rater and inter-rater reliability for each item of the PLSFRS and the consistency of the PLSFRS total score between a face-to-face examination and telephone interview using Microsoft Excel version 2210 (Microsoft Corp., Redmond, WA, USA) and IBM SPSS Statistics version 29.0 (IBM Corp., Armonk, NY, USA). Intra-rater reliability was assessed between the baseline and 8 weeks after baseline examination. Inter-rater reliability was assessed between the baseline and 4 weeks after baseline examination. The consistency between a face-to-face examination and telephone interview was assessed 4 weeks after the baseline examination.

## Results

### Demographics

Nineteen patients were enrolled at baseline. The average age of the participants at baseline was 68.2 (42–85) years. The sample included eight men (42.1%) and 11 women (57.9%). The median disease duration was 72 (12–264) months.

### Dropouts

Four participants were excluded from the study. Two patients had aspiration pneumonia during the evaluation period; however this was not considered an adverse event associated with the evaluation. The evaluation could not be completed for the other two patients because they could not consult the doctor within the evaluation period.

### Intra-rater and inter-rater reliability

The quadratic-weighted kappa coefficients for intra-rater and inter-rater agreement were substantial (intra-rater; 0.691-1.000, inter-rater; 0.634-1.000) (Table 3).

**Table 3:** Intra-rater and inter-rater consistency in this study

Items	Intra-rater κ index^†^ (95% CI)	Inter-rater κ index* (95% CI)
Language	0.976 (0.947-1.000)	0.992 (0.976-1.000)
Salivation	0.929 (0.809-1.000)	0.982 (0.956-1.000)
Swallowing	0.691 (0.319-1.000)	0.688 (0.338-1.000)
Handwriting	0.893 (0.765-1.000)	0.794 (0.511-1.000)
Cutting food and handling utensils	0.934 (0.838-1.000)	0.878 (0.744-1.000)
Dressing and hygiene	0.910 (0.815-1.000)	0.920 (0.825-1.000)
Turning in bed and adjusting bedclothes	0.849 (0.652-1.000)	0.921 (0.809-1.000)
Walking	0.951 (0.898-1.000)	0.973 (0.945-1.000)
Climbing Stairs	0.931 (0.847-1.000)	0.949 (0.889-1.000)
Dyspnea	0.906 (0.837-1.000)	0.706 (0.342-1.000)
Orthopnea	0.789 (0.766–0.811)	0.634 (0.309–0.959)
Respiratory insufficiency	1.000 (1.000–1.000)	1.000 (1.000–1.000)

^†^κ index, the quadratic weighted kappa coefficient. CI, confidence interval.

### Consistency

The intraclass correlation coefficient for the PLSFRS total score between face-to-face examination and telephone interview was 0.997 (95% confidence interval, 0.992–0.999)

## Discussion

PLS is a rare motor neuron disease, and to date, no clinical trials have been conducted. To develop therapeutic drugs in the future, it will be necessary to work globally, which includes collecting cases; therefore, creating a Japanese version of the PLSFRS is essential. Previously, the Amyotrophic Lateral Sclerosis Functional Rating Scale-Revised (ALSFRS-R) alone evaluated the intra-rater and inter-rater reliabilities. The usefulness of administering the ALSFRS-R over the telephone was subsequently evaluated [4, 5]. Telephone interviews minimized the burden on participants and caregivers by reducing the number of visits required to the study sites. Four consultations, including three visits in 8 weeks, may have increased the burden; however, no adverse events were associated with the visits for this evaluation. The availability of quick and reliable measures that can be administered over the telephone is indispensable for conducting this type of research. Thus, in this study, the consistency of the telephone interviews was ensured from the outset. Repeated evaluator training is crucial for maintaining reliability of the scale. When creating this scale, we considered the Japanese lifestyle and, added instructions to prevent confusion during the evaluation. The Japanese version of the PLSFRS is provided as a supplement, and the original instructions were referenced and added to the table for the evaluators’ convenience.

The present study has several limitations. The current diagnostic criteria require the absence of clinical and electrophysiological lower motor neuron involvement for at least 4 years after symptom onset before diagnosing PLS [6]. Although the participants in this study were recruited according to the criteria, some patients were in the early stage of disease onset and may have been diagnosed with upper motor neuron-dominant amyotrophic lateral sclerosis. Moreover, we implemented less stringent electromyography criteria to enroll more participants and accommodate a wider spectrum of patients with PLS. This approach can be an ambivalent issue because the participants who developed pure upper motor disease for less than 4 years could not receive a definite diagnosis and may have been classified as having predominantly upper motor neuron dominant amyotrophic lateral sclerosis [7]. The concern with the PLSFRS was that, since there were two additional score tiers compared to the ALSFRS-R, except for orthopnea and respiratory insufficiency, the evaluator and the participant can get confused about the subtleties. We initially believed that evaluations by phone or in person would be difficult; however, the results showed no significant differences. Therefore, the Japanese version of the PLSFRS may be a valuable indicator for assessing medical conditions, even when face-to-face assessment is impossible due to disasters or hospital visit burdens.

## Conclusions

This study provides the results for the Japanese version of the PLSFRS intra-rater and inter-rater reliability and an evaluation of the consistency between in-person and telephone interviews. Similar to the ALSFRS-R for amyotrophic lateral sclerosis, we believe that the Japanese version of the PLSFRS will be widely used not only by neurologists but also by internists and medical staff other than doctors and is a well-validated tool for PLS researchers to conduct future clinical trials.

### Electronic supplementary material

Below is the link to the electronic supplementary material.

Supplementary Material 1

## Data Availability

No datasets were generated or analysed during the current study.

## Declarations

### Previous presentations

These data were presented at the 64th Annual Meeting of Japanese Society of Neurology, Chiba, Japan, the 41st Annual Meeting of Japanese Society of Neurotherapy, Tokyo, Japan, Neurodegenerative Disease Workshop in 2020, 2023, Tokyo, Japan.

## Abbreviations

ALSFRS-R: Amyotrophic Lateral Sclerosis Functional Rating Scale-Revised
PLS: primary lateral sclerosis
PLSFRS: PLS Functional Rating Scale

## References

[1] Statland JM, Barohn RJ, Dimachkie MM, Floeter MK, Mitsumoto H. Primary lateral sclerosis. Neurologic Clin. 2015;33:749–60. 10.1016/j.ncl.2015.07.007. 10.1016/j.ncl.2015.07.007 PMC4628724 26515619

[2] Gordon PH, Cheng B, Katz IB, Pinto M, Hays AP, Mitsumoto H, et al. The natural history of primary lateral sclerosis. Neurology. 2006;66:647–53. 10.1212/01.wnl.0000200962.94777.71. 16534101 10.1212/01.wnl.0000200962.94777.71

[3] Mitsumoto H, Chiuzan C, Gilmore M, Zhang Y, Simmons Z, Paganoni S, et al. Primary lateral sclerosis (PLS) functional rating scale: PLS-specific clinimetric scale. Muscle Nerve. 2020;61:163–72. 10.1002/mus.26765. 31758557 10.1002/mus.26765

[4] Kasarskis EJ, Dempsey-Hall L, Thompson MM, Luu LC, Mendiondo M, Kryscio R. Rating the severity of ALS by caregivers over the telephone using the ALSFRS-R. Amyotroph Lateral Scler Other Motor Neuron Disord. 2005;6:50–4. 10.1080/14660820510027107. 16036426 10.1080/14660820510027107

[5] Kaufmann P, Levy G, Montes J, Buchsbaum R, Barsdorf AI, Battista V, et al. Excellent inter-rater, intra-rater, and telephone-administered reliability of the ALSFRS-R in a multicenter clinical trial. Amyotroph Lateral Scler. 2007;8:42–6. 10.1080/17482960600888156. 17364435 10.1080/17482960600888156

[6] Turner MR, Barohn RJ, Corcia P, Fink JK, Harms MB, Kiernan MC, et al. Primary lateral sclerosis: consensus diagnostic criteria. J Neurol Neurosurg Psychiatry. 2020;91:373–77. 10.1136/jnnp-2019-322541. 32029539 10.1136/jnnp-2019-322541 PMC7147236

[7] Gordon PH, Miller RG, Moore DH. ALSFRS-R. Amyotroph lateral Scler Other Motor Neuron Disord. 2004;5: 90–3. 10.1080/17434470410019906. 10.1080/17434470410019906 15512883

