# Supplementary material for: Reliability and consistency of the Japanese version of the Primary Lateral Sclerosis Functional Rating Scale
Source: BMC Neurol. 2024 Aug 13;24:282. doi: 10.1186/s12883-024-03729-6 (PMC11320838; doi:10.1186/s12883-024-03729-6)
Supplement: Supplementary file 1 — Supplementary Material 1 [file 12883_2024_3729_MOESM1_ESM.pdf]

1. Speech（言語）：被験者との会話時の状態に基づいて評価

	原文	日本語版	Instruction
6	normal speech processes	正常	-
5	occasional speech disturbance, not detectable to others	時に会話障害の自覚はあるが、自分以外には感知されていない	①舌の疲労、普段と同じ様に聞こえるように、慎重になったり、努力を要する<br>②会話障害は発語の状態を評価し、言語理解は問わない。
4	detectable speech disturbance	会話障害が認められる	自分以外も会話障害に気づいている<br>ゆっくりとしか話すことができない
3	alteration in habits to improve speech	会話が伝わりやすいように自ら何らかの工夫をしている	工夫；<br>①声がはっきりしている時に電話する<br>②水を飲みながら話す
2	intelligible with repeating	繰り返し聞くと意味がわかる	聞き直しが必要
1	speech combined with non-<br>vocal communication	声以外の伝達手段と会話を併用	伝達手段；身振り手振り、筆談、文字盤、タブレット端末など
0	loss of useful speech	実用的会話の喪失	伝達手段がなければ会話できない

2. Salivation（唾液分泌）:被験者との会話時、及び夜間/食事の際の流涎の様子に基づき評価

	原文	日本語版	Instruction
6	normal	正常	-
5	possible excess saliva, may notice increased frequency of swallowing or coughing	唾液が多めで、飲み込みや咳の頻度の増加に気づくことがある	-
4	slight but define excess of saliva in mouth, may have nighttime drooling	唾液は明らかに過剰で夜間はよだれが垂れることがある	①昼間にはよだれは垂れない<br>②起床時に枕が濡れている事がある
3	define nighttime drooling may have daytime drooling	いつも夜間にはよだれが垂れ、昼間も時によだれが垂れることがある	-
2	moderately excessive saliva, may have minimal drooling	中等度に過剰な唾液があり、よだれが垂れることがある	昼夜問わずよだれは同じ様に垂れるが、ぬぐう必要性はない
1	marked excess of saliva with some drooling	顕著に過剰な唾液があり、よだれが垂れる	会話中などによだれをぬぐう必要がある
0	marked drooling, requires constant tissue	著しいよだれで絶えずティッシュやハンカチを必要とする	ガーゼ、低圧持続吸引機を口に加えていなければならない

3. Swallowing（嚥下）：普段の食事内容、食事中の様子を聴取し評価

	原文	日本語版	Instruction
6	normal eating habits	正常な食べ方	-
5	early modification to eating habits, may have occasional gagging or coughing, no episodes of choking	何らかの食べ方が変化し、時に嘔気や咳払いがあるが、喉に詰まらせることはない	①食べ方の変化；食事時間が長くなる、摂取時体位の変化など<br>②通常より早く摂取しようとすると時に嘔気や咳払いがある<br>③自発的に咳払いしている
4	early eating problems, occasional choking	何らかの食べ方が変化し、時に食物を喉に詰まらせる	①水分（とろみ剤なし）を摂取するとむせる<br>②乾いた物などが飲み込みにくい<br>③ゆっくり食べないと詰まらせる
3	regular episodes of choking, most foods are problematic	大半の食物で喉に詰まらせるエピソードがある	①大半の食物；固形（刻み食、一口サイズ含）、半固形食、ミキサー食<br>②ゆっくり摂取しても詰まらせる
2	dietary consistency changes	食物の形態の変化が必要	①水分はとろみ剤をつけ、飲み込みにくい食物を避ける<br>②宗教的またはその他の健康上の理由は含まれない
1	needs supplemental tube feedings	補助的なチューブ栄養を必要とする	経口栄養と併用
0	NPD（exclusively parenteral or enteral feedings）	全面的に非経口性又は腸管性栄養	-

4. Handwriting（書字）：実際に被験者が書字（東西南北など）した結果に基づき評価

	原文	日本語版	Instruction
6	normal	正常	-
5	slower, but not sloppy, requires more focus	病前より遅いが、拙劣なわけではなく、病前より集中力を要する	病前と同じ字を書くためには集中力を要する
4	slow and sloppy, all words legible	遅くかつ拙劣（すべての単語は判読可能）	-
3	modifications to writing style（size, cursive versus print, etc.）	書体の変化（サイズ、筆記体とブロック体など）	①判読可能<br>②筆記用具に何らか工夫（例；太いペンなどを持つ）をしたり、画数の多い漢字の記載は避ける
2	not all words legible	一部の単語が判読不可能	-
1	able to grip pen, unable to write	ペンは握れるが字は書けない	-
0	unable to grip pen	ペンが握れない。	-

5. Cutting food and handling utensils（摂食動作）；食事用具の使い方

	原文	日本語版	Instruction
6	normal	正常	-
5	occasional difficulty cutting certain types of foods, no help needed	フォーク、スプーンや箸の扱いは自立しているが、時に特定の食物では扱いが困難 他人の助けは必要ない	特定の食物；豆類、つるつるしたもの、固いもの
4	somewhat slow and clumsy, needs no help	フォーク、スプーンの扱いは自立しているが、食物に関わらず箸の扱いは幾分時間がかかり ぎこちない 他人の助けは必要ない	-
3	using a modified method for cutting foods or handling utensils	フォーク、スプーンの扱いは自立しているが、箸は障害に合わせた用具であれば使える	他人の助けは必要ない
2	can cut most foods, slow or clumsy, some help needed	フォーク、スプーンは使えるが、箸は障害に合わせた器具を用いても使えない	ほとんどの食物は切れるが固い食物は助けが必要な時がある
1	foods cut by someone else, can still feed slowly	食物は誰かに切ってもらわなければならないが、何とかフォークやスプーンでゆっくり摂取できる	上肢装具なども用いて摂取している
0	needs to be fed	誰かに食べさせてもらわなければならない	-

6. Dressing and hygiene（着衣、身の回りの動作）

	原文	日本語版	Instruction
6	normal	正常	-
5	slower but completely independent	病前より遅いが、完全に自立している	-
4	independent self-care with effort or decreased efficiency	努力して、あるいは効率が悪いが自立している	以前と同じ手順を踏んでできない
3	modifications to the frequency and/or completion of tasks, considering the use of one or more assistive devices	着衣や身の回りの動作をする頻度かつ/又はやり方が変化し、1つ以上の介助器具の使用が考慮されている	①ひげそりが持ち続けられないのでひげをそる回数が減る<br>②下肢の疲労感があるのでシャワーの回数が減る
2	intermittent assistance or substitute methods	時折手助け又は代わりの方法が必要	①自力でも何とか服を着ることはできるが最後は介助しないと着終えることができない<br>②急ぎの時には助けが必要<br>③寝転んでパンツをはく<br>④代わりの方法：介護用の服（ボタンやジッパー、ベルトなどがない服）を着たり、ボタンの代わりにマジックテープを使用するなど
1	needs attendant for self-care	身の回りの動作に手助けが必要	①自力で服を着る事はできず途中で介助が必要<br>②シャワーに入ること、出ることは介助してもらい、体を洗うのも髪と背中は洗ってもらうが、残りの部分は自分で洗うことができる
0	total dependence	全面的に他人に依存	-

7. Turning in bed and adjusting bedclothes（寝床での動作）

	原文	日本語版	Instruction
6	normal	正常	-
5	slower, slight difficulty	病前より遅く、少し困難	ぎこちないわけではない
4	somewhat slow or clumsy, needs no help	幾分遅かったり、ぎこちなかったりするが他人の助けを必要としない	-
3	nearly always slow and clumsy, may require intermittent assistance	常に遅くぎこちない、時々介助が必要になることがある	体調の悪い日は介助が必要
2	can turn alone or adjust sheets with great difficulty	独りで寝がえりをうったり寝具を整えられるが非常に苦労する	どちらか一つだけしかできなくても該当
1	can initiate, cannot turn or adjust sheets	寝返りを始めることができるが、独りで寝返りをうったり寝具を整えることはできない	独りでは最後まで寝返りをうてず、寝具も整えることはできないので他人の助けが必要
0	helpless	自分ではどうすることもできない	-

8. Walking（歩行）；腰痛・骨折等で歩けない場合は、ありのままの状態（臨床上実際に確認できる状態）で評価

	原文	日本語版	Instruction
6	normal	正常	-
5	slow, may notice occasional tripping or change in gait	困難ではないが遅い<br>時に躓いたり、歩行が変化したりすることを自覚する	下肢の挙上が病前より悪いことを自覚している
4	early ambulation difficulty	やや歩行が困難	①休憩を挟まないと病前に可能であった距離までは歩けない<br>②補助具は必要としない、又は補助具は使用しているが、使用しなくても実際に歩行は可能
3	mixture of walking independently and with assistance	自立歩行と補助歩行の併存	間欠的に杖や歩行器又は他人の補助の併用が必要
2	walks with assistance	補助歩行	常になにかしらの補助が必要
1	non-ambulatory functional movement only	歩行は不可能	車いす移乗は可能
0	no purposeful leg movement	脚を動かすことはできない	車椅子移乗も不可能

9. Climbing stairs（階段登り）；
腰痛・骨折等で歩けない場合は、ありのままの状態（臨床上実際に確認できる状態）で評価

	原文	日本語版	Instruction
6	normal	正常	-
5	exercising caution and focus	注意と集中が必要	ふみ外したりしないように慎重になる<br>速度は低下しない
4	slow	遅い	明らかに速度は低下したが、介助は必要なく、不安定さや疲労もなし
3	intermittent unsteadiness or fatigue	軽度の不安定又は疲労	時に不安定さや疲労があるが、介助は必要なし
2	mild to moderate unsteadiness or fatigue, may use handrail	軽度から中等度の不安定又は疲労を常に感じ、手すりを使用することがある	手すりや杖を使用するが、他人の介助は必要なし
1	needs assistance	介助が必要	自力で這って登る場合も含む
0	cannot do	登れない	-

R-1 Dyspnea（呼吸困難）

	原文	日本語版	Instruction
6	none	なし	-
5	occurs occasionally with activities more strenuous than walking	歩行以上の激しい活動でたまに起きる	歩行以上；ランニング、階段登りなど
4	occurs when walking	歩行中に起きる	-
3	occurs intermittently with activities<br>other than walking	歩行以外の活動で断続的に起きる	歩行以外；日常生活（食事、入浴、着替え）のいずれかで時に苦しくなることがある
2	occurs with one or more;<br>eating, bathing, dressing	日常動作（食事、入浴、着替え）のいずれかで起こる	日常動作のいずれかで必ず苦しくなるので休みながら行う
1	occurs at rest, either sitting or lying	座位又は臥位いずれかで起きる	-
0	significantly difficultly, considering mechanical support	極めて困難で呼吸補助装置を考慮する	-

R-2 Orthopnea（起坐呼吸）

	原文	ALSFRS-R
4	none	なし
3	some difficulty sleeping, d/t shortness of breath, does not routinely use more than two pillows	息切れのため夜間の睡眠がやや困難だが、2個以上の補助寝具、介護ベット相当のものは常に必要ではない
2	needs extra pillows to sleep（>2）	眠るのに支えとなる枕や介護ベットが2個以上必要
1	can only sleep sitting up	座位でないと眠れない
0	unable to sleep	全く眠れない

R-3 Respiratory insufficiency（呼吸不全）

	原文	ALSFRS-R
4	none	なし
3	intermittent use of BiPAP	間欠的に呼吸補助装置（BiPAP）が必要
2	continuous use of BiPAP at night	夜間に継続的に呼吸補助装置（BiPAP）が必要 日中も使用しているが1日中ではない
1	continuous use of BiPAP day and night	1日中呼吸補助装置（BiPAP）が必要
0	invasive mechanical ventilation by intubation/trach	挿管又は気管切開による人工呼吸が必要

If R-3 ≦2, score R-2 as 0 (unable to sleep)
